# The Role of Family as a Source of Health Information Among College Students

**DOI:** 10.1007/s10900-025-01448-8

**Published:** 2025-02-22

**Authors:** Karthika S. Cohen, Dawn Fallik, Anne R. Cappola

**Affiliations:** 1 Penn Medical Communication Research Institute, Perelman School of Medicine, University of Pennsylvania, 3400 Civic Center Blvd, Bldg 521, Philadelphia, PA 19014 USA; 2 University of Delaware, Newark, DE USA; 3 Division of Endocrinology, Diabetes, and Metabolism, Department of Medicine, Perelman School of Medicine, University of Pennsylvania, Philadelphia, PA USA

**Keywords:** College students, Health communication, Health information, Health information Sources, Internet, Medical communication

## Abstract

The internet has increasingly become a major source of health information, especially for college-age adults, who spend a significant amount of time online. This article investigates sources used by US college students to acquire health information. College students aged 18–25 years old (*n* = 189) from 18 colleges were surveyed between November 2022 and February 2023. The survey was conducted using the online survey platform SurveyMonkey. Participants were asked to select their main sources of health information under three different categories: general medical, mental health, and Covid-19 related information. Survey data were analyzed using Microsoft Excel. The survey found that parents/guardians, the internet, and medical providers were the top information sources in each category. Although previous surveys have shown that the internet is the main source of health information for most adults, when asked about their primary source of information, college-age adults reported predominantly relying on family for general medical information. However, they turned to online sources for mental health information, with Instagram as the top social media resource. The internet was the primary source for Covid-19 information. These data suggest that including parents in health messaging for college- age adults could help with dissemination of health information to this age group particularly when addressing general medical information such as vaccines, medical care and seasonal illnesses like flu. They also suggest that this group seeks alternative sources—like peers and social media— for topics that have associated stigma, such as mental health. Providing shareable resources with mental health information through college communication programs and networks may help disseminate accurate information to students.

**Supplementary Information:**

The online version contains supplementary material available at 10.1007/s10900-025-01448-8.

## Introduction

Health information has never been easier to access. People obtain information on health, wellness, and medical topics from friends and family, television, newspapers, the internet, and social media. A 2013 Pew Research study found that the internet was a major source of health information for most Americans [1]. Nearly 60% of adult Americans surveyed said they had looked for health information on the internet in the preceding year. In Pew’s national survey in 2012, seven in ten adult internet users said they had searched the internet for information on a range of health issues, especially advice on specific diseases and treatments [2]. In 2018, a survey of 1,700 American adults conducted by the global public relations firm Weber Shandwick found that nearly three-quarters of Americans sought health information online, with over half of them seeking information online on a weekly or monthly basis [3]. Data from the Health Information National Trends Survey found that the internet was the most frequently used source of health information among American adults from 2008 to 2017, with the percentage of participants turning to the internet as a first source for their most recent health-related search ranging from 61.2% in 2008 to 74.4% in 2017 [4]. A survey by the American Health Information Management Association in October 2021 found that while 59% of Americans contacted their clinical team regarding specific medical questions or conditions, just as many turned to the Internet to find this information [5].

### Online Health Information-Seeking Behaviors

A systematic review of online health information-seeking behaviors between 2016 and 2021 found that a large majority of consumers of online health information considered the information important and impactful to their health decision making, health maintenance, and perspectives on health-related issues [6]. More than 80% of consumers used a search engine to find health information and 15% visited specific health-related websites. Social media platforms were more likely to be used for information related to stress. Topics of most interest included diet and wellness, specific health conditions and treatments, mental health, and sexual/reproductive health.

Among young Americans, a larger proportion search for health information online. In 2008, 79% of those aged 18–29 searched for health information online, more than a third looked for mental health advice on the internet, 38% turned to the internet for guidance on medications, and 34% looked for alternative treatments or drugs online [7]. Younger people are generally more adept at researching online for health information and also more satisfied with the results and quality of content they find compared with older adults. They also tend to find information online with minimal effort [8]. A study conducted among first-year college students found that 78% looked for health information online in the previous year. Similar percentages of students reported having consulted a medical professional (75.5%) and traditional media (74.6%). The same study also found that family and friends were the most popular source of health information for students, with 89.5% of participants indicating they had contacted this group for health information [9]. Surprisingly, much of the data collected about health information sourcing is dated, and few studies incorporate the explosion of social media, including TikTok, which half of college students use daily [10].

### Young Adult Sources of Health Information

In addition to using the internet for their health and wellness information, young people recognize healthcare professionals and their parents as valuable information sources. Mendes et al. conducted a qualitative study to determine the health-information-seeking behaviors of healthy young people [11]. The study determined that while healthy college students searched for health and lifestyle information online, they still recognized the unreliability of content on the internet and perceived healthcare professionals as the most trustworthy sources of health information. However, this study was conducted among medical school students at a public Portuguese university, which may have skewed the results toward more health-literate individuals. A study conducted by Vader et al. found that American college students turned most often to parents for their health information [12]. While parents were not considered the most credible sources, trailing behind health center staff, health educators, and faculty, they were nonetheless the most used sources. Since this study, health-related websites have expanded exponentially; social media has spawned online health communities; and smartphones now offer health information at the touch of a button.

A 2018 survey study conducted at a public university in New Jersey investigated the health information-seeking behaviors of students in a personal health class [13]. The study found that a majority (74%) of the 258 students surveyed were most likely to use the internet for health information over other sources. While 71% said that they were likely or very likely to review information from several web sources to assess validity and accuracy, 55% indicated that they confirmed the information they found with a healthcare provider. Among participants, women were more likely to use the internet for health information and consult a medical professional as well as confirm the health information they found with a provider. Non-White students were significantly more likely to use the internet to find health information. In a Croatian study of 469 high school students that assessed health information sources, 72% of participants identified parents as their most reliable personal source of health information and 30% indicated that the internet was their primary non-personal source of health information [14].

Much of the research related to health information-seeking among college-age adults was done prior to the Covid-19 pandemic, and many studies date back to the mid- to late 2000s when the internet was not replete with as many sources of health information as it is today and most social media platforms popular among young people did not exist. While we found many studies exploring the health information sources of adults generally, as well as some dedicated to the health information-seeking behaviors of college students in other countries, we did not find any such studies conducted in the US in the aftermath of Covid-19.

### The Present Study

In this paper, we sought to investigate the current sources that US-based, college-age adults most frequently use for critical health information, such as how to promote health and prevent disease, how to find Covid-19 related information, and how to access information about mental health. We were specifically interested in the health information-seeking behaviors of college-age adults because many individuals begin to assume independent responsibility for their health during this period. This age group has specific needs for access to health information, with higher rates of unplanned pregnancies and mortality and lower access to health care compared with those in age groups immediately younger or older [15]. Brain development is not complete until the mid- to late 20s, which may affect important health decisions. Additionally, in the US, people in this age group newly gain the right to interact with healthcare providers without parental or guardian consent.

## Methods

### Survey

The survey was created in SurveyMonkey and unique web links were sent to journalism professors around the country through select contacts. Professors were requested to send the surveys to undergraduate students in their classes. A total of 18 professors were contacted and 189 students completed the survey, of whom 133 were from a school of journalism, 52 from a liberal arts college, and 4 from a community college. An attempt was made to include diversity in the selected schools by geographic area as well as race and ethnicity. Survey participants were required to be between 18 and 25 years of age. They had the option to receive a $5 Starbucks gift card as compensation for their time. Surveys were sent out between November 2022 and February 2023. Survey links were automatically closed when the maximum number of responses (30) from each school was received. Any links still open were closed in March 2023 when final data were downloaded and analyzed.

### Measures

The survey included demographic questions to determine age, race, and ethnicity of participants. Via multiple choice questions, participants were asked to select the sources they used to obtain general medical, Covid-19, and mental health information. Source choices included parents/guardians or family, the internet, medical providers, news website, television, radio, podcast, and print publication. Participants were not asked for more specifics on the type of internet source or if the information was found through an active search or by passive content consumption. Participants were then asked what, if any, social media channels they used for information on these health-related topics and to name one primary source of information in each category. Participants were also asked about characteristics that are likely to influence individual health awareness, including if they had a chronic disease, if they considered themselves health conscious, what their parents’ education levels were, and if a parent or guardian was a healthcare provider [Appendix 1]. The survey included 24 questions and the average time for completion was 4 min.

### Informed Consent

The online survey was anonymous and did not require any identifying information from participants. It was classified exempt by the Institutional Review Board. Participants were informed of the purpose of the study, the voluntary nature of participation, and study confidentiality and anonymity.

### Statistical Analysis

Survey data were downloaded and analyzed using Microsoft Excel. Comparisons were made among the different sources of information for the three main categories of health information: general medical, Covid-19, and mental health. Sources were also analyzed for differences between participants based on race, ethnicity, and gender. Two-sample independent tests for proportions were performed to determine the significance level of the differences between each source and among the three different categories of health information.

## Results

All participants were between 18 and 25 years of age, with 74% identifying as female and 18% as male. Participants were comprised of 73% White respondents, 12% Asian respondents; and 9% Black respondents. 10% of participants identified as Hispanic (Table 1).

**Table 1:** Demographic profile of survey participants (*n* = 189)

Age, years	
Mean	20
Median	21.5
Range	18–25
Gender, n (%)	
Male	34 (18%)
Female	140 (74%)
Nonbinary/Nonconforming	4 (2%)
Prefer not to answer	11 (6%)
Race, n (%)	
AI/AN	1 (0.5%)
Asian	22 (12%)
Black	17 (9%)
NHPI	0 (0%)
White	139 (73%)
Two or more races	3 (1.5%)
Prefer not to answer	7 (4%)
Ethnicity, n (%)	
Hispanic	19 (10%)
Non-Hispanic	163 (86%)
Prefer not to answer	7 (4%)
Region, n (%)	
US-Northeast	50 (26%)
US-Midwest	30 (16%)
US-South	66 (35%)
US-West	43 (23%)

**Fig. 1:**
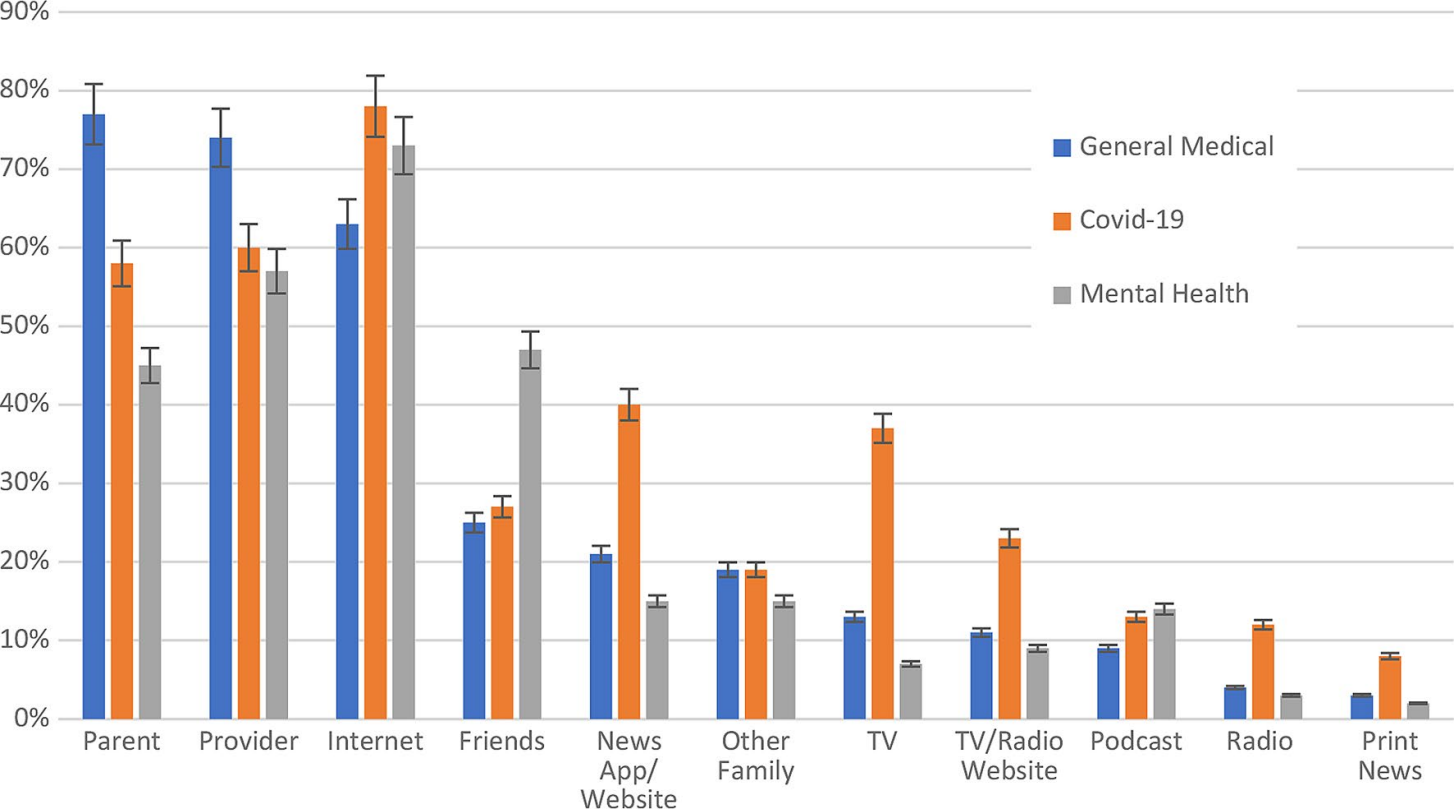
Distribution of sources for general medical, Covid-19, and mental health-related information used by young adults. Bars represent percent error. HCP = health care provider

The top sources in all categories of health information (general medical, Covid-19, and mental health) were parents/guardians, the internet, and medical providers. Participants were more likely to use parents and providers for general health information and the internet for mental health and Covid-19 information (Fig. 1).

### General Medical Information

77% of participants (141/183) obtained general medical information from their parents/guardians, 74% (135/183) from medical providers, and 63% (115/183) from general internet searches, through search engines such as Bing, Google, and Yahoo (Fig. 1). Half of participants (92/183) obtained their general health information from social media. Among those who acquired information from social media, Instagram was the top source at 85% (78/92), followed by TikTok at 64% (59/92), and YouTube at 54% (50/92).

**Fig. 2:**
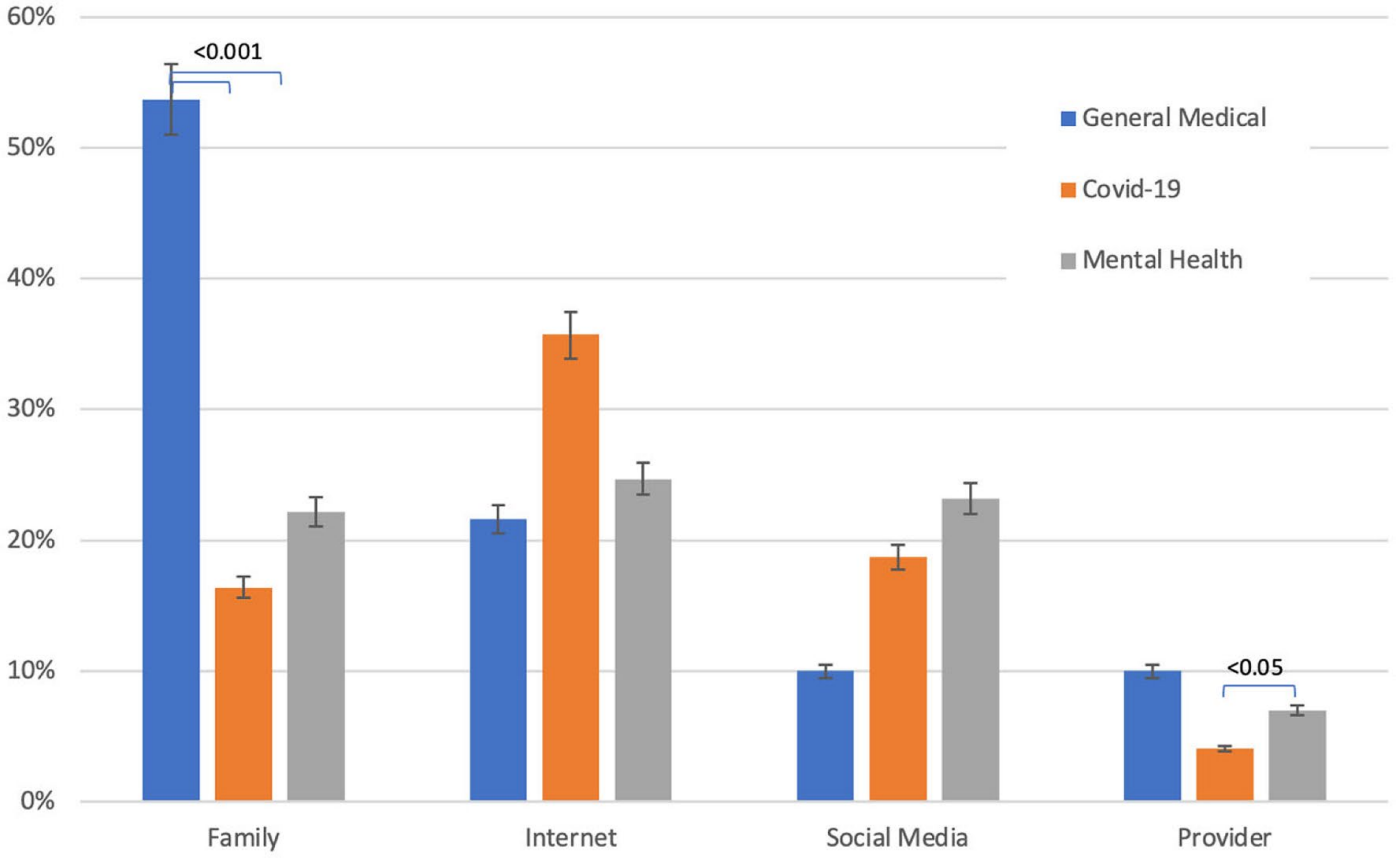
Young adults’ primary sources of general medical, Covid-19, and mental health-related information by source (family, internet, social media, and medical provider). Bars represent percent error

When participants were asked to pick just one source that qualified as their *primary* information source, “family” outranked all other sources, at 54%, with the internet as the second most common primary information source at 22% (Fig. 2). The differences were significant for the proportion of participants who used family as their primary source versus each of the other sources of information as well as for those who used the internet versus social media as their primary source of general medical information (Table 2).

**Table 2:** Young adults’ primary sources of general medical, Covid-19, and mental health-related information by source

	General Medical Information (*n* = 190)	Covid-19 Information (*n* = 171)	Mental Health Information (*n* = 198)
Family, n (%)	102 (53.7%)*	28 (16.4%)^δ^	44 (22.2%)
Internet, n (%)	41 (21.6%)**	61 (35.7%)^δδ^	49 (24.7%)
Social Media, n (%)	19 (10.0%)	32 (18.7%)^δδδ^	46 (23.2%)
Provider, n (%)	19 (10.0%)	7 (4.1%)	14 (7.0%)^φ^

One participant wrote in an additional primary source for general medical information and 9 participants wrote in an additional primary source for mental health information

* p-value < 0.001 for pairwise comparisons of family with internet, social media, and provider

** p-value < 0.001 for pairwise comparisons of internet with social media and provider

^δ^ p-value < 0.001 for pairwise comparisons of family with internet and provider

^δδ^ p-value 0.002 for pairwise comparison of internet with social media and < 0.001 for pairwise comparison of internet with provider

^δδδ^ p-value < 0.001 for pairwise comparison of social media with provider

^φ^ p-value < 0.001 for pairwise comparison of provider with family, internet, and social media

### Covid-19-Related Information

General internet search was the most predominant source (71% [125/175]) for Covid-19 related information, followed by medical provider (60% [105/175]), and parent/guardian (58% [101/175]) (Fig. 1). Social media was a source of Covid-19 related information for 69% (120/174) of participants. Of those obtaining Covid-19 related information from social media, 83% acquired it from Instagram, 71% from TikTok, and 60% from Twitter.

The internet was the primary source of Covid-19 information at 36%, followed by social media at 19% and family at 16% (Fig. 2). The proportion of participants who used internet as their primary source was significantly higher than the proportion who primarily turned to family, social media, or their provider (Table 2). The proportion of participants who used family or social media as their primary source was significantly higher than those who primarily acquired information from their providers.

### Mental Health Information

Among sources for mental health information, general internet search ranked the highest at 65% (113/175), followed by medical provider at 57% (99/175). Friends and parents/guardians as sources were similar at 47% (82/175) and 45% (79/175), respectively. 67% of respondents (116/174) obtained their mental health information from social media, compared with 33% (58/174) who did not. Instagram and TikTok again led as the most popular social media sources at 91% (107/117) and 76% (89/117), respectively.

Internet (25%), social media (23%), and family (22%) were the top three primary sources of mental health information, with the proportions of participants turning to each of the three sources being significantly higher than those obtaining mental health information from their providers (Fig. 2; Table 2).

### Analyses by Race and Ethnicity

**Fig. 3:**
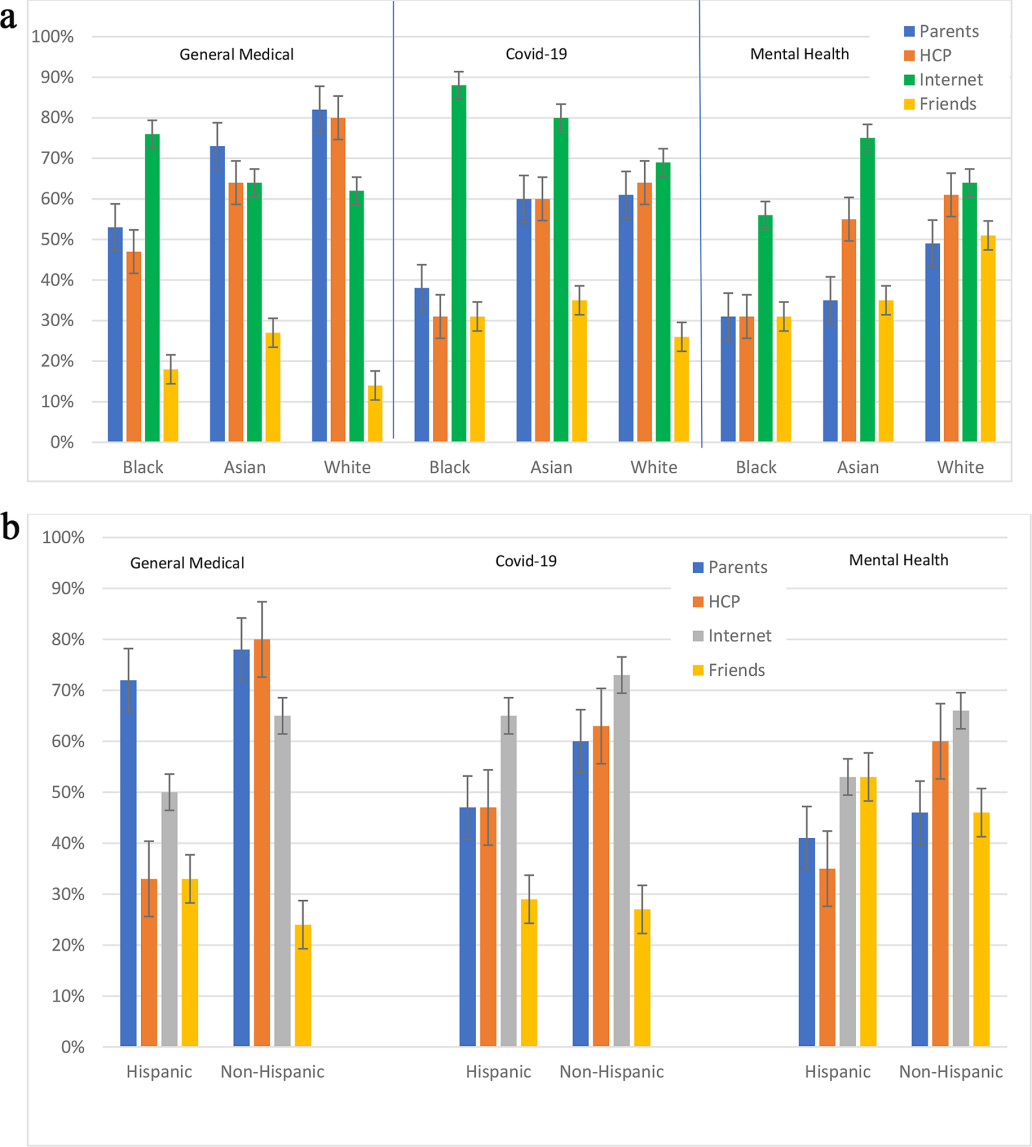
Sources of general medical, Covid-19, and mental health-related information among young adults by race (**a**) and (**b**) by ethnicity. More than one source could be selected. Bars represent standard deviation. HCP = health care provider

Differences in sources of health information based on race and ethnicity are shown in Fig. 3. For general medical and Covid-19 information, Black respondents relied more on the internet than other sources. For mental health information, the internet was the predominant source for all races except for White respondents, who relied almost equally on healthcare providers. However, none of these results reached statistical significance.

## Discussion

We found that college-age adults used their parents as their primary source of general medical information, whereas the internet and social media played larger roles as sources of information about Covid-19 and mental health. We were unable to find previous work showing that college-age individuals use different sources for different types of health information. Our data also suggest that there may be differences in accessing parents and healthcare providers as sources of health information by race and ethnicity.

Vader et al.’s study sought to determine US college students’ sources for health information by asking them to rate their most believable sources for such information [12]. However, the survey, administered in 2011 using the American College Health Association–National College Health Assessment database, was conducted before the Covid-19 pandemic and at a time when online sources for health information were not as expansive as they currently are. Notably, the survey focused only on the four “most believable” sources of health information as informed by survey participants, which did not include online sources. The survey found that health center medical staff, health educators, faculty or coursework, and parents were the most believable sources—in that order (though printed media and the internet were included in the list of choices, among others). Consistent with our results, parents were reported to be the most used source of health information. The study found that those who turned to their parents for health information tended to be White, younger, female, and single. The survey also found that these perspectives had remained fairly consistent since 2000. While racial differences were not statistically significant in our study, likely due to small sample size, we found that White participants relied more heavily on parents for general medical information than other races.

The survey study by Basch et al. found that the internet was the most likely source of health information for college-age adults [13]. However, this work was also conducted prior to the Covid-19 pandemic, and hence did not account for the shifting paradigm of health information and misinformation. Moreover, participants selected their preferred health sources via a Likert scale, so the study did not identify primary sources; it also did not differentiate among different categories of health information.

Our finding that this age group shows a strong reliance on the internet to obtain health information is consistent with previous, older surveys. Escoffery et al. performed a survey study to gauge the online health information-seeking behavior of American college students. The study reported that 73% (542/743) got health information online and the most common method to find health information was a search engine. 15% of students in the study said they had used the internet for health information in the preceding week and 32% in the previous month [16]. With regard to the ability to find information, 11% reported always being able to find the information they were pursuing, and 56% found it most of the time. Only 25% talked to a provider about the information they found online. However, this study did not draw comparisons between use of the internet versus other sources to obtain health information.

A narrative literature review from 2021 that investigated use of digital technologies for health from ages 5 to 30 years found that younger people relied on websites and search engines to obtain health information [17]. Younger people desired more knowledge about their bodies and health, better ways to improve health and fitness, and the ability to connect with peers for emotional and mental support on platforms like YouTube and online forums. The study also found that youth continued to look to trusted adults to comprehend and interpret the information they found online and as additional sources of information and support—a finding corroborated by our study—with a preference for face-to-face interactions with such trusted figures.

While our study found that reliance on medical providers was high, when asked exclusively for their *primary* source of health information, the proportion of participants choosing provider as a source was low, possibly because participants in this demographic neither have easy access to clinicians nor a high frequency of office visits with clinicians [18]. This group would also be aging out of their pediatric care facilities and possibly in transition between providers. Additionally, the Covid-19 pandemic may have reduced clinical visits, as it did in much of the population. Another reason for lower reliance on providers may be that patients do not always leave a provider’s office with all their questions answered, prompting them to seek answers online [19]. Additionally, individuals in this age group may be more comfortable with other sources such as friends and the internet. This was seen especially in the case of mental health information, where nearly half the participants used friends and nearly three quarters used the internet as information sources. The percentage of participants getting mental health information from friends was nearly double the percentage of those who obtained general medical information from friends.

Confidentiality and privacy are major concerns for this age group, and one of the incentives for seeking out information online. Making independent choices as adults while still depending on parents for emotional and financial support is unique to this group, leading to complicated healthcare decisions. In a 2019 study involving semi-structured interviews with 20–23 year-old women insured through their parents’ health insurance plans, many participants indicated their preference for autonomy over personal health information, in some cases even paying out of pocket or using free health clinics in order to keep health information private from parents [20].

It is important to consider health inequities that may be propagated by procurement of information online. Previous work focused on the online behavior of younger populations has found that even among digital natives, gender, race, and socioeconomic status may influence how the internet is used to find relevant information. A 2010 study of first year college students found that being White, Asian, or male, or having parents with higher education levels were associated with better web skills [21]. Additionally, higher socioeconomic status predicted how well people incorporate and use the internet in their daily lives. More affluent individuals used the internet in better informed ways for a variety of different uses and activities. However, a follow-up study found that the online health information-seeking behavior of college students varied more based on online skills and internet experience than on their race, gender, or socioeconomic status [22]. The amount and quality of information young people find on the internet can vary widely based on their abilities to search for content online.

In this age of exploding online health information, digital health literacy is key to navigating the vast troves of information on the internet. Higher health literacy corresponds to healthier behaviors [23]. The ability to navigate health information online can thus lead to health disparities [21]. A study conducted among a representative population of 256 US college students found that higher levels of self-reported digital health literacy, but not health literacy, were significantly associated with greater compliance with COVID-19 vaccine recommendations and acceptance of the negative impacts of acquiring the disease [24].

A survey conducted among US college students in the immediate aftermath of the Covid-19 pandemic investigated the online Covid-19 information sources used by participants and tested their knowledge about the virus. The majority of participants indicated using the internet and social media “most often” and “always” to obtain Covid-19 information in comparison with news publications, radio, and television. Survey respondents also identified family and friends, health professionals, and schools/universities as health information sources “most often” and “always” used versus books/brochures or the faith community. More than a third of participants (*n* = 394/992) indicated that the internet was where they had heard the most information on Covid-19. Based on answers to the health literacy questions, most participants had a basic knowledge of the disease, but few, that is 18% (*n* = 173/966) were able to answer all questions about Covid-19 symptoms correctly [25]. Studies such as these emphasize the need to improve digital and health literacy across this age group.

### Limitations

Our study was limited by the relatively small number of participants. With 74% of participants being White and female, the sample was not ethnically- or gender-diverse. However, the participant pool was geographically diverse, with representation across US regions. All participants were students in a journalism course, which may affect generalizability. It is possible that journalism students may conduct more online research or consume more social media content than other adults in this age group. Overlapping categories for sources of health information among multiple-choice questions, such as internet and social media, news websites and newspapers, and news websites and the internet may have skewed results. Multiple choice questions with the ability to select multiple answers may have produced redundancies, which we attempted to resolve by asking participants to name a primary source.

### Study Implications

College students are digital natives and hence may be more susceptible to erroneous health information due to their strong dependence on the internet for information consumption. To address these vulnerabilities, the focus should be on countering health misinformation online, especially on social media and other platforms frequented by young people. Health authorities, medical professionals and communicators, and health reporters should work to bust myths pervading the internet by preemptively debunking conspiracy theories, opportunistically introducing evidence-based facts into the news cycle, and nimbly responding to high-impact misinformation. Social media platforms also have a responsibility to combat misinformation by flagging erroneous content and suspending accounts that spread them.

It is concerning, though not surprising, that this age group prefers to acquire mental health information from the internet and friends rather than from mental health providers and medical professionals. Adults with mental health conditions are especially dependent on the internet for information, despite the knowledge that online information can be unreliable [26]. Young people lean on internet sources to understand mental health issues, find better therapies, learn about adverse reactions to medications, as well as to prepare for doctor’s office visits and challenge what they hear from providers, as determined by a study among those aged 18–30 [27].

For these reasons, it is imperative that mental health care be made more easily accessible to everyone, especially youth, by reducing cost and transportation barriers, providing telehealth services, and alleviating privacy concerns. Additionally, since this group seeks mental health advice from friends, more efforts must be made to provide peers with shareable resources that present accurate and comprehensible mental health information to reduce the chance of spreading misinformation. Colleges, fitness centers, and healthcare facilities can find ways to supply accurate information to this group via seminars, online videos, and printed materials. Proactive methods to deliver important health service announcements to this population are essential, since pandemic fatigue and greater negative affect can contribute to avoidance of information regarding major public health concerns due to feelings of hopelessness and anxiety [28].

Improving health literacy is key to improving healthy behaviors [23]. It is during transition to college that healthy lifestyle behaviors may change, including eating habits, alcohol use, physical activity, and weight maintenance. Studies have shown that improving healthy behaviors at this age leads to better overall health and fitness, both concurrently and later in life [29]. Hence, it is important that this population accesses accurate information, inoculating it against misinformation and conspiracies, to encourage well-informed health choices.

## Conclusion

The observation that individuals enrolled in college rely heavily on parents and family for health information reinforces the need to educate parents, so that they are better able to communicate accurate information to their dependents and children, given the low health literacy levels among US adults [30]. Provision of up-to-date health information through healthcare facilities including community clinics and health fairs, health promotion campaigns that focus on parent-child communication, instruction on explaining complex health issues, as well as newsletters, webinars, or parent orientation/training can help educate adults and their dependents.

## Electronic Supplementary Material

Below is the link to the electronic supplementary material.

Supplementary Material 1
