# Supplementary material for: The Role of Family as a Source of Health Information Among College Students
Source: J Community Health. 2025 Feb 22;50(4):613–22. doi: 10.1007/s10900-025-01448-8 (PMC12301270; doi:10.1007/s10900-025-01448-8)
Supplement: Supplementary file 1 — Supplementary Material 1 [file 10900_2025_1448_MOESM1_ESM.docx]

Welcome! **Thank you for participating in our survey. The purpose of the survey is to determine where college students get their medical/health, mental health, and COVID-19- related information. The survey has a total of 24 questions and should take roughly**

**5 minutes to complete; the anonymous information will be used for research.**

**By completing this survey, you are consenting to participate in this study. No personally identiﬁable information will be collected in the survey. Once you complete and submit the survey, you will receive a link where you can provide an email address to receive a $5 gift card from Starbucks.**

**The survey creators will not have access to your email address or any other personal information.**

1. Age


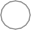
 18
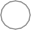
 23


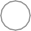
 19
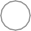
 24


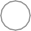
 20
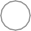
 25


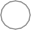
21
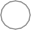
 None of the above


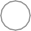
22

1. Gender


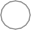

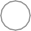
 Male
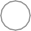
 Female

Transgender


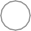
 Non-binary


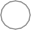

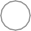
 Non-conforming Prefer not to answer

1. Race


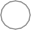
 American Indian or Alaska Native
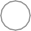
 Asian or Asian American


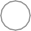
 Black


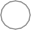
Other (please specify)


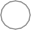
 Native Hawaiian or Other Paciﬁc Islander
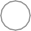
 White


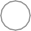
Prefer not to answer

1. Are you of Hispanic or Latino origin?


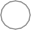
 Yes
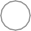
 No


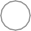
 Prefer not to answer

1. Name of school
2.
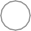

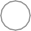

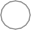

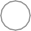
Year started college

| 2014 | 2019 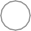 |
| --- | --- |
| 2015 | 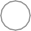 2020 |
| 2016 | 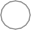 2021 |
| 2017 | 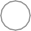 2022 |
| 2018 | None of the above 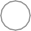 |

1.
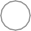
Highest education level of parent/guardian (Please choose parent/guardian with the highest education level)


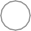

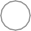
 Did not complete high school
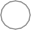
 High school/GED

Bachelor's


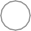
 Master's


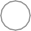

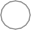
 Advanced degree Prefer not to answer

* 8. Is a parent or guardian a healthcare provider?


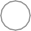

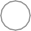
 Yes No

1. Please indicate type of healthcare provider


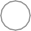
 Physician
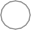
 Nurse


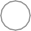
 Nurse practitioner
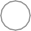
 Physician assistant

1. Do you live full time at home (with parents/guardians)?


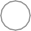
 Yes
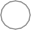
 No


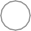
 Prefer not to answer

1. Where do you get your general medical/health information? Select all that apply.

Parent/Guardian Other family Friends

Medical provider

General internet search (Bing, Google, Yahoo etc.) Newspaper app or website

Television or radio website Television news (network or cable) Radio

Podcast

Print Newspaper

Other (please specify)


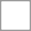
 None of the above

1. Do you get your medical/health information from social media?


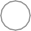

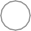
 Yes No

1. Which social media channel do you use? Select all that apply.

Instagram TikTok Snapchat YouTube

Facebook Twitter Whatsapp

Other (please specify)

1. Which of the above sources—including family, friends, the internet, radio, television, newspaper, and social media—is your primary source of medical/health information?
2. Where do you get mental health information? Select all that apply.

Parent/Guardian Other family Friends

Medical provider

General internet search (Bing, Google, Yahoo etc.) Newspaper app or website

Television or radio website Television news (network or cable) Radio

Podcast

Print newspaper

Other (please specify)


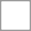
 None of the above

1. Do you get your mental health information from social media?


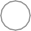

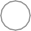
 Yes No

1. Which social media channel do you use? Select all that apply

Instagram TikTok Snapchat

YouTube Facebook Whatsapp

Other (please specify)

1. Which of the above sources—including family, friends, the internet, radio, television, newspaper, and social media—is your primary source of mental health information?
2. Where do you get your COVID-19-related information? Select all that apply.

Parent/Guardian Other family Friends

Medical provider

General internet search (Bing, Google, Yahoo etc.) Newspaper app or website

Television or radio website Television news (network or cable) Radio

Podcast

Print newspaper

Other (please specify)


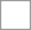
 None of the above

1. Do you get your COVID-19-related information from social media?


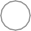

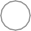
 Yes No

1. Which social media channel do you use? Select all that apply.

Instagram TikTok Snapchat YouTube

Facebook Twitter Whatsapp

Other (please specify)

1. Which of the above sources—including family, friends, the internet, radio, television, newspaper, and social media—is your primary source of COVID-19-related information?
2. Does your college provide adequate access to health, medical, and dental information and resources?


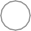
 Yes
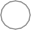
 No


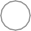
 Don't know


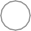
 Other (please specify)

1. Who makes your doctor’s appointments? Select all that apply.


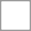
 Self


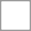
 Parent or Guardian
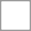
 Prefer not to answer

Other (please specify)

1. Do you have a chronic health condition that requires regular medical follow-up?


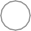
 Yes
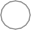
 No


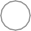
 Prefer not to answer

1. Would you consider yourself a health-conscious person?


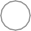
 Yes
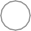
 No


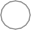
 Maybe


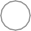
 Please provide additional comments, if needed.

Thank you for taking the survey! **Please head to the link below to ﬁll out your email address so that a $5 Starbucks gift card can be emailed to you. The URL is unique to each individual, so please do not share.**

[**https://www.surveymonkey.com/r/M2G3PWS**](https://www.surveymonkey.com/r/M2G3PWS)
